# Use of medical services and medicines attributable to type 2 diabetes care in Yaoundé, Cameroon: a cross-sectional study

**DOI:** 10.1186/s12913-017-2197-0

**Published:** 2017-04-11

**Authors:** Clarisse Mapa-Tassou, Leopold K. Fezeu, Zakariaou Njoumemi, Eric Lontchi-Yimagou, Eugène Sobngwi, Jean Claude Mbanya

**Affiliations:** 1 Department of Public Health, Faculty of Medicine and Biomedical sciences, The University of Yaoundé I, Yaoundé, Cameroon; 2 University of Paris 13, Sorbonne Paris Cité – UREN (Unité de Recherche en Epidémiologie Nutritionnelle), U557 Inserm; U1125 Inra; Cnam; CRNH IdF, F-93017 Bobigny, France; 3 Diabetes Research and Training Centre and Division of Endocrinology, Department of Medicine, Albert Einstein College of Medicine, Bronx, New York USA; 4 Department of Internal Medicine and specialties, Faculty of Medicine and Biomedical sciences, The University of Yaoundé I, Yaoundé, Cameroon

**Keywords:** Type 2 diabetes, Complications, Medical services, Medicines, Cameroon, Africa

## Abstract

**Background:**

The increasing numbers of people with type 2 diabetes (T2D) is a global concern and especially in sub-Saharan Africa, where diabetes must compete for resources with communicable diseases. Diabetes intensifies health care utilisation and leads to an increase in medical care costs. However, In Cameroon like in most developing countries, data on the impact of diabetes on the medical health system are scarce. We aimed to analyse the use of medical services and medicines attributable to T2D care in Yaoundé, Cameroon.

**Methods:**

We conducted a cross-sectional study comparing the use of medical services and medicines on 500 people with T2D attending the diabetic outpatient units of three hospitals in Yaoundé and 500 people without diabetes matched for age, sex and residence. We performed multivariate logistic and quantile regressions to assess the effect of diabetes on the use of medical services and medicines and the presence of other chronic health problems. Models were adjusted for age, educational level, marital status, occupation and family income.

**Results:**

Overall, the rate of use of health services was found to be greater in people with T2D than those without diabetes. People with T2D had greater odds of having an outpatient visit to any clinician (OR 97.1 [95% CI: 41.6–226.2]), to be hospitalised (OR 11.9 [95% CI: 1.6–87.9]), to take at least one medicine (OR 83.1 [37.1–185.8]) compared with people without diabetes. We also observed an association between diabetes and some chronic diseases/diabetes complications including hypertension (OR 9.2 [95% CI: 5.0–16.9]), cardiovascular diseases (OR 1.9 [95% CI: 0.8–4.9]), peripheral neuropathy (OR 6.2 [95% CI: 3.4–11.2]), and erectile dysfunction (OR 5.8 [95% CI: 2.7–12.1]).

**Conclusions:**

This study showed that the presence of diabetes is associated with an increased use of health care services and medicines as well as with some chronic diseases/diabetes complications.

**Electronic supplementary material:**

The online version of this article (doi:10.1186/s12913-017-2197-0) contains supplementary material, which is available to authorized users.

## Background

Diabetes is a challenging problem for public health worldwide with an estimated 415 million people or 8.8% of adults aged 20–79 affected in 2015–75% of whom live in low- and middle-income countries. With current trends, it has been projected that about 642 million people, or one adult in ten, will have diabetes 2040 [1]. The progression of diabetes is part of a broader epidemiological transition from communicable to non-communicable diseases [2–8]. An estimated 14.2 million adults aged 20–79 have diabetes in Africa, representing a regional prevalence of 2.1–6.7%. Africa has the highest proportion of undiagnosed diabetes; over two thirds (66.7%) of people with diabetes are unaware they have the disease [1]. Moreover, Africa is facing a health time bomb with diabetes having an increasing impact on people of working age; more than three quarters of deaths due to diabetes in 2015 occurred in people under the age of 60 [1]. The burden of diabetes in sub-Saharan Africa is already substantial, and patients with this condition make significant demands on health care resources [9]. In Cameroon, diabetes was not considered a public health priority until after the results of a 2003 epidemiological study revealed that diabetes was a growing potential threat to health [10]. In 2015, the prevalence of diabetes was 5.3% in the 20 to 79 years age group with more than 14,998 diabetes-related deaths [1]. This transition has been attributed to the population aging, increasingly sedentary lifestyles and obesity [1, 2]. With the ongoing population aging, it is expected that Africa will continue to experience an increase in the number of patients as well as those with related comorbidities. This is also associated with considerable consequences for health care and related costs [11–18]. The costs associated with diabetes usually include the increased use of health services, loss of productivity and disability. Diabetes therefore imposes a large economic burden on individuals and families, national health systems and nations as a whole, making the disease a significant obstacle to sustainable economic development. Data on the use of resources for diabetes care in sub-Saharan Africa are particularly scarce, difficult to generalize and sometimes outdated, although they all indicate that the diabetes-caused use of medical services and medications is substantial [19–22].

The African Diabetes Impact Study was initiated in sub-Saharan Africa because of the paucity of data on the socio-economic cost of diabetes and the inadequacy of national policies for the prevention and control of diabetes. A Pan African multicentre study was initiated with the purpose of increasing knowledge on type 2 diabetes, with focus on socio-economic aspects. In this study, we aimed to analyse the use of medical services and medicines attributable to T2D care in Yaoundé, Cameroon.

## Methods

### Design

We conducted a cross-sectional study, comparing the use of medical services and medicines in people with T2D and people without diabetes.

### Sample selection

#### Sample size and statistical power

Data to calculate statistical power for an economic study in sub-Saharan Africa are not available. However, applied to the formula for matched samples [23], results from a similar recently completed study in Shanghai [13] suggest that 500 cases and 500 controls will yield statistically significant results. This sample size also gives > 90% power to detect a difference in proportions of 10 percentage points or larger at an alpha of 0.05.

#### Recruitment of people with type 2 diabetes

People with T2D were recruited consecutively from outpatient diabetes clinics at three different hospitals in Yaoundé: the Yaoundé Central Hospital, where 80% of cases were obtained, the Biyem-Assi District Hospital, and the Cité Verte District Hospital. We consecutively interviewed 500 people with T2D attending the outpatient diabetes units of these three hospitals.

#### Recruitment of people without diabetes

In order to obtain participants without diabetes comparable to the participants with diabetes, we asked the latter to identify five people living in their neighbourhood who were comparable to them by age (+/− 5 years) and sex. We therefore contacted and recruited one control subject for each case using this information. Potential controls who said they had been diagnosed with diabetes were excluded. One completed control interview was obtained for each selected person with T2D. All participants were aged between 20 and 80 years.

### Data collection

Trained field workers conducted identical structured face-to-face interviews with all participants in their homes to ascertain recent use of medical care services, medicines and the presence of other chronic diseases using a questionnaire (Additional file 1).

### Estimation of the use of medical services

The study team relied on participants recall to ascertain the use of medical services. To increase accuracy, we limited recall to a 90-day window and, to calibrate the window, we asked respondents to name and associate a well-remembered event that occurred approximately 90 days ago. In order to obtain information on the use of medicines, interviewers asked participants to show to them the medicines they were using. To further increase accuracy, we asked for details only about the most recent hospital outpatient visit, overnight hospitalization, and purchase of each medicine. The study team then used the descriptions of this subset of the most recent events to estimate the characteristics of all events of the same kind, such as reasons for admissions and visits and mean length of hospital stay. Although participants were asked about visits to hospital emergency wards, very few of such visits were reported to support analysis. We therefore excluded data on emergency-ward visits from this report. The recall window for hospitalizations was 365 days. We also enquired about other chronic health conditions such as known complications of diabetes (kidney diseases; erectile dysfunction or loss of libido; amputation of toes, foot, or leg; peripheral neuropathy; depression, eye diseases) and others (cardiovascular diseases, cancer, lung diseases, HIV/AIDS).

### Statistical analyses

Interview responses were recorded on the paper interview form and subsequently entered into an electronic data base created using the EPI Data version 3.1 software. Prior to analysis, we cleaned variables in the database and discrepancies were then re-checked against the hard-copy records.

Results are expressed as means ± SD or median (25th – 75th percentiles) and frequencies (percentages) for quantitative and qualitative variables respectively. For univariate analyses, comparisons were made between people with diabetes and people without diabetes using paired Student t tests for continuous variables; McNemar test was used for qualitative variables with fewer than three modalities and a Stuart-Maxwell test for qualitative variables with at least three modalities.

Multivariate logistic and quantile regression models were used to assess the effect of diabetes on the use of medical services, medicines and the presence of other chronic health problems, from which odds ratios (OR) or median regression coefficient were calculated as well as their corresponding 95% confidence intervals (CI) or their standard errors where appropriate. Models were adjusted for age, educational level, marital status, occupation and family income.

Statistical tests were two-sided. Results were interpreted as significant only when *p*-value were <0.05. Statistical analyses were performed using Stata software (version 13.1®, Stata Institute).

### Ethical considerations

Ethical clearance was obtained from the National Ethics Committee for Health Research on Humans of the Ministry of Public Health of Cameroon. Written informed consent was obtained for all participants enrolled.

## Results

### Demographics

Table 1 shows socio-demographic characteristics of the study population. People with T2D and people without diabetes did not differ significantly in terms of gender and Family income. People with T2D were 1.1 years older than people without diabetes (56.2 ± 10.7 years vs. 55.1 ± 10.6 years, *p* < 0.001). Compared to people without diabetes, people with T2D were more educated, unemployed and had a bigger family. They were also more often single and a small number of them were smokers. For people with diabetes, the median duration of diabetes was 5 (25th–75th percentiles: 3–10) years.

**Table 1:** Socio-demographic characteristics of study participants

Characteristics	People with T2D	People without diabetes	*p*-value
Mean age, mean ± SD	56.20 10.7	55.11 ± 10.6	<0.001*
Age classes, n (%)			<0.001**
≤ 40	36 (7.2)	52 (10.4)	
41–50	119 (23.8)	117 (23.4)	
51–60	174 (34.8)	183 (36.6)	
61–70	121 (24.2)	118 (23.6)	
> 70	50 (10)	30 (6)	
Gender, n (%)			1***
Male	241 (48)	241 (48)	
Female	259 (51.8)	259 (51.8)	
Diabetes duration, years median (1^st^–3^rd^)	5 (3–10)	---	---
Diabetes duration, years			
0–5	268 (53.6)		
6–10	98 (19.6)		
≥ 11	134 (26.8)		
Educational level, n (%)			<0.01**
Primary	238 (47.6)	246 (49.2)	
secondary	190 (38)	208 (41.6)	
University	72 (14.4)	46 (9.2)	
Marital status, n (%)			<0.001***
Not living in couple	175 (35)	158 (31.7)	
Living in couple	325 (65)	341 (68.3)	
Occupation, n (%)			<0.001***
Working	227 (45.4)	279 (55.8)	
Not working	273 (54.6)	221 (44.2)	
Not working because of ill, n (%)	36 (7.2)	3 (0.6)	<0.001***
Family size, mean (SD)	6.81 (3.6)	5.59 (2.9)	<0.001*
Family income, USD/person/year median (1^st^-3^rd^) ^a^	2400 (1440–4800)	2400 (1380–3600)	>0.05*
Tobacco smoking, n (%)			<0.001**
Never smoked	400 (80)	342 (68.4)	
Ex-smoker	85 (17)	49 (9.8)	
Current smoker	15 (3)	109 (21.8)	

*p-values from paired *t*-test; **p-values from Stuart-Maxwell test and ***p-values from McNemar test

^**a**^Family income was available only for 204 persons with diabetes and 173 persons without diabetes. Mean annual family income per person is the monthly respondent’s self-reported family income times 12 months.1 USD = 500 XAF. *USD* United States Dollar

### Use of outpatient services

Table 2 depicts the self-reported use of medical services. About 90% of people with T2D and only 12% of those without diabetes reported having made at least one outpatient hospital visit in the last 90 days (*p* < 0.001). The 90-day median for total visits, among people with T2D, with at least one visit, was 3 (25th – 75th percentiles: 2–4) and 1 (25th – 75th percentiles: 1–3) visits for people without diabetes (*p* < 0.001).

**Table 2:** Use of medical services and medicines by the study population

Use of services	People with T2D	People without diabetes	OR (95% CI)^a^	median (95% CI)	*p*-value
Use of outpatient services					
Estimated percentage of people with at least 1 visit to hospital, last 90 days, n (%)	485 (97)	70 (14)	267.6 (83.9–854.2)	---	<0.001
Estimated percentage of people with at least 1 outpatient visit to hospital, last 90 days, n (%)	454 (90.8)	60 (12)	129.8 (58.1–289.9)	---	<0.001
Median outpatient visits at the hospital per person, among those with at least 1 outpatient visit during the last 90 days, median (1^st^-3^rd^)	3 (2–4)	1 (1–2)	---	1.5 (0.7–2.3)	<0.01
Estimated percentage of people with at least 1 outpatient visit to any clinician, last 90 days, n (%)	460 (92)	98 (19.6)	97.1 (41.6–226.2)	---	<0.001
Median total outpatient visits to any clinician, last 90 days, median (1^st^-3^rd^)	3 (1–4)	0 (0–0)	---	2 (1.8–2.2)	<0.001
Use of inpatient services					
Estimated percentage of people with at least 1 inpatient admission, last 90 days, n (%)	45 (9)	2 (0.4)	10.9 (1.5–81.6)	---	<0.05
Median length of stay, most recent hospital admission, if admitted last 90 days, median (1^st^-3^rd^)	8 (5–15)	2 (1–3)	---	9.17 (−28.2–9.9)	NS
Estimated annual percentage of people with at least 1 inpatient admission, n (%)	47 (9.4)	3 (0.6)	11.9 (1.6–87.9)	---	<0.05
Use of medicines					
Estimated percentage of people with at least one medicine, n (%)	468 (93.6)	114 (22.8)	83.1 (37.1–185.8)	---	<0.001

^a^The OR and the median regression were computed with adjustments on age, gender, education and income

Considering visits to any clinician, people with T2D had 97 times more odds to have a visit than those without diabetes (OR 97.1 [95% CI: 41.6–226.2], *p* < 0.001). Among people with T2D, outpatient visits did not vary significantly by age or by duration of diagnosed diabetes (all *p* > 0.05).

### Use of inpatient services

Table 2 shows the use of inpatient services by participants in the two groups. Nine percent of people with T2D reported at least one admission during the preceding 90 days, compared to 0.4% in the non-diabetic group (OR 10.9 [95% CI: 1.5–81.6], *p* < 0.05).

The median hospital length of stay, among those admitted during the preceding 90 days, was 8 (25th–75th percentiles: 5–15) for people with T2D and 2 (25th–75th percentiles: 1–3) for those without diabetes. Moreover 47 people with T2D spent at least one night at the hospital during the past year and therefore had 11 times more odds of being hospitalised compared to people without diabetes (OR 11.9 [95% CI: 1.6–87.9], *p* < 0.05).

### Reasons for inpatient admissions and outpatient visits

Table 3 shows the reasons for hospital admissions and hospital outpatient visits. About half (53.3%) of the hospital admissions of people with T2D was attributed to diabetes, 6.7% to heart diseases and 2.2% to eye diseases. People with T2D attributed 91.5% of their most recent hospital outpatient visits to diabetes, while those without diabetes attributed 75.4% of their visits to other diseases.

**Table 3:** Self-reported causes of inpatient admissions and outpatient visits

Reason	Inpatient admissions	Hospital outpatient visits	
	People with T2D n (%)	People with T2D n (%)	People without diabetes n (%)
Diabetes	24 (53.3)	455 (91.5)	0 (0)
Lung diseases	5 (11.1)	0 (0)	2 (3.3)
Heart diseases	3 (6.7)	6 (1.2)	9 (14.7)
Eyes diseases	1 (2.2)	7 (1.4)	3 (0.6)
Kidney diseases	0 (0)	3 (0.6)	1 (1.6)
Trauma	0 (0)	1 (0.2)	0 (0)
Other	12 (26.7)	7 (1.4)	46 (75.4)

### Use of medicines

Nearly all people with T2D (93.6%) possessed and reported that they were on at least one medication at the time they were interviewed, compared to one-fifth (22.8%) of people without diabetes, (OR 83.1 [95% CI: 37.1–185.8]), *p* < 0.001. Moreover the use of medicines increased with age and with the duration of diabetes (all *p* < 0.05).

Among people with T2D, 89.6% were on a glucose lowering agent, 41.6% on an antihypertensive treatment, 3.2% on a lipid lowering agent, and 5.6% on daily aspirin.

### Chronic health problems associated with diabetes

The Table 4 presents the associations between T2D and other chronic health problems. We observed correlations between diabetes and some chronic health diseases. People with T2D had more odds of having hypertension (OR 9.2 [95% CI: 5.0–16.9]), cardiovascular diseases (OR 1.9 [95% CI: 0.8–4.9]), peripheral neuropathy (OR 6.2 [95% CI: 3.4–11.3]), and erectile dysfunction (OR 5.8 [95% CI: 2.7–12.2]) compared to those without diabetes (*p* < 0.01). More than 86% of people with diabetes had at least one comorbidity or complication. No statistical association was observed between the proportions of cancer, HIV/AIDS, lung, kidney and eyes-related diseases in the two groups.

**Table 4:** Health chronic problems declared by the study population

Chronic diseases	People with T2D n (%)	People without diabetes n (%)	*p*-value*	OR (95% CI)**	*p*-value**
Hypertension	250 (50.1)	79 (15.8)	<0.001	9.2 (5.0–16.9)	<0.001
Cardiovascular diseases	47 (9.4)	21 (4.2)	<0.01	1.9 (0.8–4.9)	<0.01
Peripheral neuropathy	271 (54.2)	117 (23.5)	<0.001	6.2 (3.4–11.3)	<0.001
Erectile dysfunction or loss of libido	143 (29.3)	52 (10.4)	<0.001	5.79 (2.7–12.2)	<0.001
Foot or leg ulcer/Amputation	30 (6)	9 (1.8)	<0.001	2.5 (0.7–8.4)	>0.05
Kidney diseases	39 (7.8)	18 (3.6)	<0.01	0.8 (0.3–2.3)	>0.05
Eyes diseases	49 (9.8)	21 (4.2)	<0.001	1.6 (0.7–3.4)	>0.05
Depression	25 (5)	7 (1.4)	<0.01	7.6 (1.4–65.9)	>0.05
Cancer	5 (1)	3 (0.6)	0.7	-	-
Lung diseases	28 (5.6)	27 (5.4)	1	0.8 (0.3–2.1)	>0.05
HIV/AIDS^a^	5 (1)	6 (1.2)	1	0.8 (0.2–3.8)	>0.05
Comorbidity number			<0.001***		
0	68 (13.6)	194 (38.8)			
1	122 (24.4)	174 (34.8)			
≥ 2	310 (62)	132 (26.4)			

*p-value from paired *t*-test; **OR and *p*-value from multivariate logistic regression; ***p-value from the Stuart-Maxwell test

^a^HIV/AIDS: Human Immunodeficiency Virus infection and Acquired Immune Deficiency Syndrome

## Discussion

Diabetes imposes a large economic burden on the national healthcare system. The estimation of the impact of T2D on the use of health services and medicines by people with T2D is an indicator of this burden. In Cameroon, there is a limited data on the impact of T2D on the healthcare system. In this study, in order to assess the use of medical services, medicines and the presence of chronic health conditions, we conducted a cross-sectional study comparing the outcomes in a group of 500 people with T2D and 500 people without diabetes in Yaoundé, matched for age (+/− 5 years), sex and residence.

People with T2D and people without diabetes did not differ on gender and residence. People with T2D were 1.1 years older than people without diabetes. We observed a statistically significant difference in the matching criterion age which probably results from the frequency matching using 5-year age bands. In order to adjust for socio-demographic characteristics, analyses were adjusted for age, educational level, marital status, occupation and family income.

People with T2D had 97 times more odds of having an outpatient visit to any clinician, 11 times more odds of being hospitalised and 83 times more odds of being on at least one medicine, compared with people without diabetes. Considering the proportions, 92% of people with T2D reported at least one outpatient visit during the preceding 90 days, compared to 19.6% of people without diabetes. Also, 9.4% of people with T2D reported at least one inpatient admission compared to 0.4% for people without diabetes. Finally, with regards to the use of medicines, 93.6% of people with T2D were on at least one medication compared to 22.8% for people without diabetes.

These proportions are much higher than those observed in a study using a similar interview schedule design in China, where the proportions from people with T2D were 46.1% for outpatient visits, 3.6% for inpatient days and 66.2% for medicines; compared to 23.3%, 1.6% and 18.3% respectively for people without diabetes [13]. Also, in a multi-centric study in Africa including Cameroon and 3 other countries (Mali, Tanzania and South Africa), the overall proportions were also as high as in Cameroon; the proportions from people with T2D were 90.5% for outpatient visits, 4.9% for inpatient visits, 94.8% for ongoing medication; compared to 15.4, 1.1 and 26.6% respectively for people without diabetes [12].

Several factors could explain these differences. One important factor is that unlike in a comparable study in China where diabetes was ascertained by a population-based household screening, a majority of diabetes cases remain undiagnosed in Africa [24]. In Africa, testing for diabetes usually occurs after patients present with complications known to arise from diabetes [24]. This means that, as a group, people recognized as having diabetes in Africa are much sicker than their counterparts in many other places, and need more services and medication.

The under-utilization of medical services by the general population is another cause of the big differences we report. Medical care in Africa is often very expensive relative to income [24]. People with diabetes may receive more drugs because their condition brings them into frequent contact with the health care system, increasing both the likelihood of detecting and treating previously undiagnosed diseases and the continuation of medication for known chronic disorders.

A third cause is the fact that people with T2D were recruited from well-organized diabetes clinics located in the capital, with a high concentration of general practitioners, internists and diabetologists. Findings would likely to be different if people with diabetes from other parts of the country were included in the study.

Furthermore, diabetes was associated with several comorbidities. We observed associations between diabetes and other chronic conditions such as hypertension, cardiovascular diseases, peripheral neuropathy, and erectile dysfunction. This probably increases the burden on people with diabetes. Indeed, it is known that the number of comorbidities is a strong predictor for the volume of medical health care utilisation [20, 24–27].

This study has some limitations. Firstly, as it is neither possible nor desirable to affect diabetes experimentally, our research design was observational. However, our cross-sectional study with a comparison group is the most widely used approach and accepted measure of the economic and social impacts of diabetes [11, 14, 15, 28–30]. Secondly, the sample consisted of consecutive users of the outpatient diabetic clinics of hospitals in the capital city, a procedure that probably over-selected people with diabetes who were more ill and more likely to use and more likely to be able to afford the services. Also, the self-reported information raises the likelihood of recall bias but we tried to limit this by considering 3 months for outpatient visits and 1 year for hospitalization. Literature in industrialized countries suggests that patients can remember occurrences and many details of meetings of medical care for those no older than 3 months, and remember the occurrence and even hospitalization details for up to 1 year [25–27, 31, 32]. To the extent that the patient cannot recall, it does usually develop in the direction of under-reporting, particularly during outpatient visits [31, 32]. Any underreporting in our data should have the effect of skewing downwards the estimates of the effects of diabetes on the use of medical care, because the people with T2D had significantly more encounters than people without diabetes.

## Conclusion

In conclusion, this study showed that, people with T2D in Cameroon use more resources (outpatient visits, hospitalizations and medicines) than people without diabetes. Although non-communicable diseases like diabetes are commonly thought to be minor problems for health systems and patients in Africa compared to communicable diseases, there can be no doubt that diabetes imposes a significant burden on families and the health care system in Cameroon. This therefore mandates that the attention of policymakers should be drawn and actions informed by evidence should be considered.

## Additional file

Additional file 1: African DM Study impact questionnaire18. Illness Impact interview. This study compared people with and without type 2 diabetes from the same neighbourhoods matched by age and sex. Data was obtained by personal interviews using the Diabetes Impact questionnaire developed by the International Diabetes Federation for Africa. Except for a limited number of questions about diabetes treatment applicable only to the group of patients with diabetes, both groups responded to identical questions. These were divided into 4 categories as follows: Health Utility Index 3, Use of medical services, Impact of health problems and Medications. This study focuses only on the component related to the use of medical services. Most of the questions elicited information on respondents’ use of health services (outpatient visits and hospitalizations) during the period of 3 months leading up to the study as well as any medicines they were taking at the time of the interview. We also asked questions about any prevailing health conditions. (PDF 110 kb)
