# Supplementary material for: Use of medical services and medicines attributable to type 2 diabetes care in Yaoundé, Cameroon: a cross-sectional study
Source: BMC Health Serv Res. 2017 Apr 11;17:262. doi: 10.1186/s12913-017-2197-0 (PMC5387297; doi:10.1186/s12913-017-2197-0)
Supplement: Additional file 1: — African DM Study impact questionnaire18. Illness Impact interview. This study compared people with and without type 2 diabetes from the same neighbourhoods matched by age and sex. Data was obtained by personal interviews using the Diabetes Impact questionnaire developed by the International Diabetes Federation for Africa. Except for a limited number of questions about diabetes treatment applicable only to the group of patients with diabetes, both groups responded to identical questions. These were divided into 4 categories as follows: Health Utility Index 3, Use of medical services, Impact of health problems and Medications. This study focuses only on the component related to the use of medical services. Most of the questions elicited information on respondents’ use of health services (outpatient visits and hospitalizations) during the period of 3 months leading up to the study as well as any medicines they were taking at the time of the interview. We also asked questions about any prevailing health conditions. (PDF 110 kb) [file 12913_2017_2197_MOESM1_ESM.pdf]

ILLNESS IMPACT INTERVIEW

Study Code (usually, Country)	SC	Interviewer code	IC
Region/Province	L1	Checked by:	CK
District	L2	Data Entry Clerk code:	DE1
City/Town/Village	L3	2nd Data Entry Clerk:	DE2
Ward/Street	L4	Case=1, Control =2:	CASE
Language Code	LANG	Surrogate? Y=1, N=0	SUR
**Respondent Telephone:**	TEL	**Respondent ID:**	ID
**Respondent Family Name**(Surname):	FN		
**Respondent Personal Names:**	PN		

DOI: **Date of interview:** |__|__|- |__|__|- |__|__| DOI
Day Month Year

STIM: **Starting time:** |__|__| |__|__| STIM
24 hour clock
Hour Minutes

LOC: **Location of Residence** **1** Capital city / **2** Other city / **3** Town / **4** Countryside / **5** Isolated countryside LOC

SEX: **1** Male/ **2** Female SEX

MAR: **Marital status** **1** Single / **2** Married / **3** Widowed / **4** Separated or Divorced / **5** Cohabitating / **66** Other MAR

AGE: **Age in years or midpoint of range:** |__|__|__| AGE

DOB: **Date of Birth:** |__|__|- |__|__|- |__|__|__|__| DOB
Day Month Year

Enter 01 if day not know and/or if month not known

DM1: **Has a doctor or health practitioner ever told you that you have diabetes?** **1** Yes / **2** No / **8** Don't Know / **9** Refused DM1

TERM: **IF RESPONDENT IS A CONTROL AND ANSWERS YES, TERMINATE STUDY** **1** Terminate / **2** Continue TERM

WRK: Which of the following statements best describes your work situation? **1** Yes / **2** No / **8** Don't know / **9** Refused

WORK TYPE	RESPONSE
I work only around the home stead (childcare, cooking, cleaning, take care of livestock etc.)	WRK1
I am a small scale farmer	WRK2
I am self-employed but not as a farmer	WRK3
I am on formal employment (for salary or wages from a single employer) in the public sector	WRK4
I am on formal employment in the private sector (includes NGOs)	WRK5
I am not working because I am not able to work due to my health condition	WRK6
I am not working and looking for work	WRK7
I am retired or a pensioner	WRK8

WTH: Does your household own any of the following items?
1 Yes / **2** No / **8** Don't know / **9** Refused
Note: Household means all the people that eat together with the respondent

ITEMS OWNED	RESPONSE
Car	WTH1
Motorcycle	WTH2
Bicycle	WTH3
Refrigerator	WTH4
TV set	WTH5
Computer	WTH6
Fixed line telephone	WTH7
Mobile/cellular phone	WTH8
Micro-wave	WTH9
Electric cooker	WTH10
House connected with electricity	WTH11
Piped water	WTH12
Flush toilet	WTH13
Stone/brick walls	WTH14
Roofing tiled house	WTH15

INET: How many people normally eat together with you where you live? |__|__| INET

INCM: Taking those who eat with you as your household, what is your household's monthly income from all sources?
Income includes all money and goods from all sources.
(**8** Don't know/ **9** Refused) |__|__|__|__|__|__|__|__| INCM

SCH: **What is the highest level of school that you have completed?**
1 No formal school of any kind / **2** Dropped out in Primary school / **3** Finished Primary school / **4** Dropped out in Secondary school / **5** Finished Secondary school / **6** Trade school or apprenticeship (e.g., hair dressing, mechanic) / **7** Dropped out of University / **8** Finished University

SCH

HEALTH UTILITY INDEX

Read**: Now I want to ask about various aspects of your health. When answering these questions we would like you to think about your health and your ability to do things on a day to day basis, during the past week. You may feel that some of these questions do not apply to you, but it is important that we ask the same questions of everyone.**

Read**: To define the 1 week period, please think about what the date was 7 days ago and recall the major events that you have experienced during this period. Please focus your answers on your abilities, disabilities and how you have felt during the past 1 week. There are no rights or wrong answers; what we want is your opinion about your abilities and feelings.**

VISION / Read the entire sentence, emphasizing the words in italics. DO NOT read the response options.

HU1: **During the past week, have you been able to see well enough to read ordinary newsprint [or Bible or Qu'ran] *without* glasses or contact lenses?** **1** Yes (***Go to HU4***) / **2** No / **3** Don't know / **4** Refused

HU1

HU2: **Have you been able to see well enough to read ordinary newsprint [OR BIBLE OR QU'RAN] with glasses or contact lenses?** **1** Yes (***Go to HU4***) / **2** No / **3** Don't know / Did not wear glasses or contact lenses / **4** Refused

HU2

HU3: **During the past week, have you been able to see at all?**
1 Yes / **2** No (***Go to HU6***) / **3** Don't know / **4** Refused

HU3

HU4: **During the past week, have you been able to see well enough to recognize a friend on the other side of the street *without* glasses or contact lenses?** **1** Yes (***Go to HU6***) / **2** No / **3** Don't know / **4** Refused

HU4

HU5: **Have you been able to see well enough to recognize a friend on the other side of the street with glasses or contact lenses?** **1** Yes / **2** No (***Go to HU6***) / **3** Don't know/ Did not wear glasses or contact lenses / **4** Refused

HU5

HEARING

HU6: **During the past week, have you been able to hear what is said in a group conversation with at least three people *without* a hearing aid** **1** Yes (***Go to HU11***) / **2** No/ **3** Don't know / **4** Refused

HU6

HU7: **Have you been able to hear what is said in a group conversation *with* at least three other people with a hearing aid?** **1** Yes ***(Go to HU9***) / **2** No / **3** Don't know/didn't wear a hearing aid / **4** Refused

HU7

HU8: **During the past week, have you been able to hear at all?** **1** Yes / **2** No (***Go to HU11***) / **3** Don't know /**4** Refused

HU8

HU9: **During the past week, have you been able to hear what is said in a conversation with one other person in a quiet room *without* a hearing aid?** **1** Yes (***Go to HU11***) / **2** No/ **3** Don't know/ **4** Refused

HU9

HU10: **Have you been able to hear what is said in a conversation with one other person in a quiet room *with* a hearing aid?** **1** Yes /**2** No / **3** Don't know/didn't wear a hearing aid /**4** Refused

HU10

SPEECH

HU11: **During the past week, have you been able to be understood *completely* when speaking your own language with people who do not know you?** **1** Yes (***Go to HU16***) /**2** No / **3** Don't know /**4** Refused

HU11

HU12: **Have you been able to be understood *partially* when speaking with people who do not know you** **1** Yes/ **2** No/ **3** Don't know / **4** Refused

HU12

HU13: **During the past week, have you been able to be understood *completely* when speaking with people *who know you well*?** **1** Yes (***Go to HU16***) /**2** No / **3** Don't know / **4** Refused

HU13

HU14: **Have you been able to be understood *partially* when speaking with people who know you well?** **1** Yes (***Go to HU16***) / **2**.No / **3** Don't know / **4** Refused

HU14

HU15: **During the past week, have you been able to speak at all?** **1** Yes / **2** No / **3** Don't know / **4** Refused

HU15

GETTING AROUND

HU16: **During the past week, have you been able to bend, lift, jump and run *without difficulty* and *without help* or *equipment* of any kind?**
1 Yes (***Go to HU24***) / **2** No / **3** Don't know / **4** Refused
HU16

HU17: **Have you been able to walk around the neighborhood *without difficulty* and *without help* or *equipment* of any kind?**
1 Yes (***Go to HU24***) / **2** No / **3** Don't know/ **4** Refused
HU17

HU18: **Have you been able to walk around the neighborhood *with difficulty* but *without help* or *equipment* of any kind?**
1 Yes (***Go to HU24***)/ **2** No/ **3** Don't know/ **4** Refused
HU18

HU19: **During the past week, have you been able to walk at all**
1 Yes/ **2** No (***Go to HU22***)/ **3** Don't know/ **4** Refused
HU19

HU20: **Have you needed mechanical support, such as braces or a cane or crutches, to be able to walk around the neighborhood?**
1 Yes/ **2** No/ **3** Don't know/ **4** Refused
HU20

HU21: **Have you needed the help of another person to walk?**
1 Yes/ **2** No/ **3** Don't know/ **4** Refused
HU21

HU22: **Have you needed a wheelchair to get around the neighborhood?**
1 Yes/ **2** No/ **3** Don't know/ **4** Refused
HU22

HU23: **Have you needed the help of another person to get around in the wheelchair?**
1 Yes/ **2** No/ **3** Don't know / **4** Refused
HU23

HANDS and FINGERS

HU24: **During the past week, have you had the *full use* of both hands and ten fingers?**
1 Yes (***Go to HU28***)/ **2** No/ **3** Don't know/ **4** Refused
HU24

HU25: **Have you needed the help of another person because of limitations in the use of your hands or fingers?**
1 Yes/ **2** No (***Go to HU27***)/ **3** Don't know/ **4** Refused
HU25

HU26: **Have you needed the help of another person with some tasks, most tasks, or all tasks?**
1 Some tasks/ **2** Most tasks/ **3** All tasks/ **4** Don't know **5** Refused
HU26

HU27: **Have you needed special equipment, for example special tools to help with dressing or eating, because of limitations in the use of your hands or fingers**
1 Yes/ **2** No/ **3** Don't know/ **4** Refused
HU27

SELF CARE

HU28: During the past week, have you been able to eat, bathe, dress and use the toilet without difficulty?
1 Yes (***Go to HU31***)/ **2** No/ **3** Don't know/ **4** Refused

HU28

HU29: Have you needed the help of another person to eat, bath, dress or use the toilet?
1 Yes / **2** No / **3** Don't know / **4** Refused

HU29

HU30: Have you needed special equipment or tools to eat, bathe, dress or use the toilet?
1 Yes/ **2** No/ **3** Don't know / **4** Refused

HU30

FEELINGS

HU31: During the past week, have you been feeling happy or unhappy?
1 Happy/ **2** Unhappy (***Go to HU33***)/ **3** Don't know/ **4** Refused

HU31

HU32: Would you describe yourself as having felt:
1 Happy and interested in life (***Go to HU34***)/ **2** Somewhat happy (***Go to HU34***)/ 3 Don't know/ 4 Refused

HU32

HU33: Would you describe yourself as having felt:
1 Somewhat unhappy/ **2** Very unhappy / 3 So unhappy that life is not worthwhile/ 4 Don't know/ **5** Refused

HU33

HU34: During the past week, did you ever feel fretful, angry, irritable, anxious or depressed?
1 Yes/ **2** No (***Go to HU37)*** **3** Don't know /**4** Refused

HU34

HU35: How often did you feel fretful, angry, irritable, anxious, or depressed?
1 Rarely/ **2** Occasionally / **3** Often/ **4** Almost always/ **5** Don't know/ **6** Refused

HU35

HU36: During the past week did you feel *extremely* fretful, angry, irritable, anxious or depressed; to the point of needing professional help?
1 Yes/ **2** No/ **3** Don't know/ **4** Refused

HU36

MEMORY

HU37: How would you describe your ability to remember things during the past week?
1 Able to remember most things/ **2** Somewhat forgetful/ **3** Unable to remember anything at all/ **4** Don't know/ **5** Refused

HU37

THINKING

HU38: **How would you describe your ability to think and solve day to day problems, during the past week?**
1 Able to think clearly and solve problems/ **2** Had a little difficulty/ **3** Had some difficulty/ **4** Had a great deal of difficulty/ **5** Unable to think or solve problems/ **6** Don't know / 7 Refused

HU38

PAIN AND DISCOMFORT

HU39: **Have you had trouble with pain or discomfort during the past week?**
1 Yes/ **2** No ***(GO TO HU41)*** / **3** Don't know / **4** Refused

HU39

HU40: **How many of your activities, during the past week were limited by pain or discomfort?**
1 None/ **2** A few/ **3** Some/ **4** Most/ **5** All/ **6** Don't know / **7** Refused

HU40

HU41: **Overall, how would you rate your health during the past week?**
1 Excellent/ **2** Very good/ **3** Good/ **4** Fair/ **5** Poor/ **6** Don't know/ **7** Refused

HU41

USE of MEDICAL SERVICES

Read**: I now want to ask you about your use of health services over the last three months. Please start by thinking of an event that happened about three months ago. Most of my questions will be about health services that you have used since that time. Can you remember something that you did or that happened to you about 3 months (90 days) ago?**

HV90: **During the LAST 90 DAYS, did you ever visit a HOSPITAL of any kind to get any kind of medical care for yourself, including just seeing a doctor or nurse or just to get medication.**
1 Yes / **2** No **(SKIP TO NONHOS)**/ **8** Don't know **(SKIP TO NONHOS)**/ **9** Refused
IF NO, MAKE SURE NO HOSPITAL VISIT OF ANY KIND, even just a visit to see a nurse, doctor or just to get medication

HV90

AD90: **During the last 90 days, how many times did you go to a hospital and end up spending at least 24 hours in a bed or stretcher?**
Enter number of times admitted to hospital/ **00** No admissions/ **8** Don't know / **9** Refused

AD90

EW90: **During the last 90 days, how many times did you go to a hospital and end up spending less than 24 hours in a bed or stretcher, for example in an observation room, emergency room, or casualty room?**
Enter number of times admitted to hospital/ **00** No admissions/ **8** Don't know / **9** Refused

EW90

OP90: **During the last 90 days, how many times did you go to a hospital to see a doctor or a nurse as an outpatient (not an emergency room visit)**

Enter number of outpatient visitsto a hospital/ **00** No outpatient visits/ **8** Don't know / **9** Refused

OP90

OP91: **During the last 90 days, how many times did you go to a hospital to get medications?**

Enter number of visits to a hospital to get medication/ **00** No visits to hospital to get medication/ **8** Don't know/ **9** Refused

OP91

AD12: **Before the times you visited a hospital during the last 90 days, how many OTHER times during the last YEAR did you spend at least one night in a hospital?**

Enter number of admissions/ **00** No admissions/ **8** Don't know/**9** Refused

AD12

Read: **Now I would like to ask about what happened during some of these visits to hospitals. Let's start with your most recent [hospital admission → observation room admission → outpatient visit. (*If no visit within last 90 days or last year, skip to NONHOS*)**

USE OF HOSPITALS	Most Recent Overnight Admission	Most Recent Observation Room or emergency room	Most Recent Routine Outpatient Visit
**Name of hospital of most recent overnight admission.** Write **00** if no overnight admission	HNAD		
**Name of hospital of most recent observation room visit**<br>Write **00** if no observation room visit	HNOP		
**Name of hospital of most recent routine outpatient visit**<br>Write **00** if no routine outpatient visit	HNEW		
**Can you remember the approximate date that you went?** DDMMYY<br>Enter 99 for any part not recalled	ADDT	EWDT	OPDT
**Which type of health facility was it?**<br>**1** Public/ **2** Private for profit/ **3** Private charity	TYAD	TYEW	TYOP
**What level of hospital is this?**<br>**1** National referral / **2** District hospital / **3** Level-3 / **4** Level-4 / **66** Other	LVAD	LVEW	LVOP

**What problem caused you to go to the hospital this time?**<br>Probe for the **primary** reason for visit: **1** Heart disease/ **2** Stroke/ **3** Kidney disease/ **4** Eye disease/ **5** Leg or foot ulcer/ **6** Cancer/ **7** Lung disease/ **8** Trauma/ **9** Diabetes/ **10** childbirth/ **66** Other/ **44** don't know	P1AD	P1EW	P1OP
**Other reason, if any.** **99**=if no secondary reason, otherwise codes as above	P2AD	P2EW	P2OP
**Other reason, if any.** **99**=if no secondary reason, otherwise codes as above	P3AD	P3EW	P3OP
**IF ADMITTED FOR LESS THAN 24HOURS: How many hours did you spend at the hospital?**<br>Enter as decimal, e.g., 1.5 = 1 hour and 30 minutes/ Enter **99** if not recalled		DAEW	DAOP
**IF ADMITTED: How many nights did you spend in a ward?**<br>Enter number of nights/ **8** Don't know or can't remember/ **9** Refused	NIWA		
**IF ADMITTED: How many nights did you spend in ICU (intensive care unit)?** Check to see that ward nights + ICU nights = total nights.<br>Enter number of nights/ **8** Don't know or can't remember/ **9** Refused	NICU		
**Did a doctor see you or only a nurse?** **1** doctor / **2** nurse / **8** Don't Know	MDAD	MDEW	MDOP
**IF SAW A DOCTOR: How MANY specialist doctors or surgeons?**<br>**99** if did not see any doctor / **8** do not know	SPAD	SPEW	SPOP
**IF SAW A DOCTOR: How MANY primary care doctors?** **99** if did not see any doctor /**8** do not know	PCAD	PCEW	PCOP
**What tests or procedures were done?** Enter codes for **ALL** that occurred.<br>**1** blood test / **2** urine test / **3** x-ray or ultrasound / **4** major surgery / **5** minor surgery / **66** other	TSAD	TSEW	TSOP
**Did you receive any pills, injections or infusions?**<br>Enter codes for **ALL** that occurred. **1** Pills / **2** Injections / **3** IV drips / **66** Other	MEAD	MEEW	MEOP
**How long did it take to get to get to this hospital this time?**<br>Enter hours as decimal, e.g., 1.5 = 1 hour and 30 minutes/ **8** Don't know	TRAD	TREW	TROP
**How did you get to the hospital?** **1** walk / **2** bicycle / **3** scooter or motorcycle / **4** public bus or van / **5** private car / **6** taxi / **7** ambulance / **66** other / **8** don't know/ **9** refused	MOAD	MOEW	MOOP
**How many people went with you on this trip?**<br>Enter number of people/ **00** if patient went alone	PEAD	PEEW	PEOP
**How much did it cost to travel to the hospital?**<br>Enter ONE WAY amount in local currency for all persons who made this trip/ 777777 if cannot recall	TCAD	TCEW	TCOP

**How much did you and your family pay for unofficial costs that were not issued a receipt during this hospital visit?** 777777 If cannot recall	PYAD	PYEW	PYPC
**How much did you and your family pay for everything that happened at this hospital visit?** (Include cost of medicine, tests done on you, food, fees paid to doctors and laboratory costs, and **exclude travel costs**) / 777777 If cannot recall	PYAD	PYEW	PYOP

NONHOS: Read **Now I want to ask about health care you received at places other than a hospital. Again, I only want to know about care you received within the last 90 days since [mention anchor event]. During the last 90 days, did you seek health care services from other places other than a hospital?**

1 Yes (*FILL OUT NON-HOSPITAL VISITS TABLE*)/ **2** No ***(GO TO NCD)*** / **8** Don't know ***(GO TO NCD)/*** **9** Refused

NONHOS

Non-Hospital Visits	**How many visits?** (LAST 90 DAYS)	**Type** (1 public / 2 private / 3 charity or NGO)**	**Travel Time** (ONE WAY, most recent visit, decimal hours)	**Cost of travel** (ONE WAY, most recent visit)	**Total fees and charges*** (most recent visit)
**Specialist Doctor or Surgeon**	SP90	SPTY	SPTT	SPTC	SPCO
**Primary Care Doctor**	PC90	PCTY	PCTT	PCTC	PCCO
**Nurse**	RN90	RNTY	RNTT	RNTC	RNCO
**Traditional Healer, including herbalists and fortune tellers, magicians, oracles**	TH90	THTY	THTT	THTC	THCO
**Pharmacist or Dispensary**	PH90	PHTY	PHTT	PHTC	PHCO
**Visit to a clinic to collect medication**	CL90	CLTY	CLTT	CLTC	CLCO
**Health Educator, such as a diabetes educator**	HE90	HETY	HETT	HETC	HECO
**Community Health Worker**	CH90	CHTY	CHTT	CHTC	CHCO

*Total fees and charges include all payments made by the respondent or the family for the medical **care that the respondent received**. Include any payments made after the visit. Include all payments for drugs, supplies, procedures and tests received. Do not include payments made by insurance, employers, or other organizations.
If patient reports having done MORE THAN one visit of a particular type, record information for the **most recent of these visits.

NCD: **I will now read a list of health conditions that people sometimes have. Please tell me which ones a doctor has EVER said you have.** **1** Yes / **2** No / **8** Don't know/ **9** Refused

Chronic health conditions	Response
Heart attack	NCD1
Heart Failure	NCD2
Other heart disease	NCD3
Stroke	NCD4
High blood pressure	NCD5
Cancer	NCD6
Asthma	NCD7
Other lung disease	NCD8
HIV/AIDS	NCD9
Erectile dysfunction or loss of libido	NCD10
Kidney disease	NCD11
Amputation of toe, foot, or leg	NCD12
Eye surgery	NCD13
Peripheral neuropathy (pain, tingling, or numbness in legs or feet)	NCD14
Foot or leg ulcer (sore that will not heal on its own)	NCD15
Laser treatment on your eyes	NCD16
Kidney dialysis	NCD17
Depression	NCD18
Other mental condition	NCD19
Other permanent problem you still have	NCD20

INF: **Now I want to ask about other temporary health problems that you have had. Within the last 90 days, since [mention anchor event], have you had…**

1 Yes/ **2** No/ **8** Don't know/ **9** Refused

Time-limited conditions	Response
Active Tuberculosis	TD1
Acute Episode of Malaria	TD2
Influenza (Flu)	TD3
Pneumonia	TD4
Diarrhea lasting 3 or more days or requiring drip or pills	TD5
Pregnancy or childbirth	TD6
Other major infection	TD7
Other major parasite	TD8
Injury	TD9
"Cold" or other virus	TD10
Typhoid	TD11
Cholera	TD12
Other temporary illness	TD13

TST: **During the LAST 90 DAYS (3 months), did someone perform any of these kinds of tests on you, including in a hospital? Do not count tests you did yourself**
1 Yes / **2** No / **8** Don't know / **9** Refused**.**

Diabetes-Related Tests	Response
Any urine test	TST1
A test on your blood taken by needle	TST2
A finger-stick blood test	TST3
Blood pressure measurement (cuff on arm)	TST4
An eye exam	TST5
A test of your feet for feeling	TST6
Taking your weight on a scale	TST7
Measuring your waist with a tape	TST8

IMPACT of HEALTH PROBLEMS

Now I would like to ask you how your health problems are affecting your life and the life of your family. Are YOUR health problems.

For questions about respondent: **1** Yes / **2** No / **8** Does Not Apply/Don't Know/ **9** Refused

For questions about family members: **1** mother / **2** father / **3** brother / **4** sister / **5** girl child / **6** boy child / **7** girl grandchild / **8** boy grandchild / 10 husband/ 11 wife/ **66** other

In this table, family is NOT limited to persons with whom the respondent eats but also includes anyone related by blood or adoption.

	Respondent	Family Member	Family Member	Family Member
Preventing you from doing *any* paying work?	IR1			
Keeping you from doing *as much* paying work as you would like?	IR2			
Making you do *more* paying work than you otherwise would?	IR3			
Preventing some other person in your family from doing *any* paying work?		IF1A	IF1B	IF1C
Keeping some other person in your family from doing *as much* paying work as they would like?		IF2A	IF2B	IF2C
Making other family members do *more* paying work than they would like?		IF3A	IF3B	IF3C

Preventing you from growing *any* food or doing *any* work in the house?	IR4			
Preventing some *other* member of your family from growing any food or doing *any* work in the house?		IF4A	IF4B	IF4C
Keeping you from doing *as much* farming or housework as you would like?	IR5			
Preventing some *other* member of your family from doing as much farming or housework as they would like?		IF5A	IF5B	IF5C
Preventing you from enrolling in school or training?	IR6			
Preventing some other member of your family from enrolling in school or training?		IF6A	IF6B	IF6C
Keeping you from seeing friends or family?	IR7			
Keeping you from getting enough to eat?	IR8			
Preventing any other member of your family from getting enough to eat?		IF8A	IF8B	IF8C

MISS: **During the LAST 90 DAYS, how many days did you miss your usual activities (work, work at home or school) because of health reasons?**
Write number of days/ **8** Don't Know/ **9** Refused

Number of days: |__|__| MISS

HIRE: **Have you or your family hired someone to take care of you because of your ill health?**
1 Yes / **2** No / **8** Don't know or remember / **9** Refused

HIRE

PAY: **IF YES, How much do you pay this person each month?**
Use leading zeros /**8** don't know**/ 9** Refused

|__|__|__|__|__|__|__|__|__|__| PAY

FCG: **Does someone from your family who is not paid take care of you because of your ill health?**
1 Yes / **2** No/ **8** Don't know or remember/ **9** Refused

FCG

FCGT: **IF YES, How much of this person's day is spent taking care of you?**
1: 1 to 25% / **2**: 26 to 50% / **3**: 51 to 75% / **4** 76 to 100%

FCGT

BTA: **During the past 12 months, how did you pay for or get the medical services, care-givers, medicines, and medical supplies that you, yourself, used?**
1 Yes / **2** No / **8** Don't know/ **9** Refused

Sources of funds	Response
By spending out of your current HOUSEHOLD income	BTA1
Social welfare support	BTA2
Donations of medicines or supplies by employers or agencies	BTA3
With money or fundraising from friends or family who live with you or near you	BTA4
With money or fundraising from family living abroad	BTA5
From saved money, such as bank account savings	BTA6
By borrowing money	BTA7
By selling possessions like furniture, animals, or jewelry	BTA8
By selling housing or land	BTA 9

ACC: **Now I am going to read you a list of reasons why people sometimes do not get medical services when they want them. Please tell me which if any of these have prevented you from getting medicines or medical care, over the last 12 months:** **1** Yes/ **2** No/ **8** Don't know/ **9** Refused

REASONS	RESPONSE
Lack of funds to pay for the care	ACC1
Lack of transportation	ACC2
No one to help me get there	ACC3
Would have taken too long to go there/too far away	ACC4
Would have had to wait too long once I was there	ACC5
Did not have health insurance	ACC6
Too sick to make the trip	ACC7
No doctor or other professional was available	ACC8
Medicines were not in stock	ACC9
Did not trust the care	ACC10
Did not know where to go	ACC11
Some other reason	ACC12

SMOK: **Do you smoke cigarettes, sniff or chew tobacco pipes or cigars?** **1** Yes/ **2** No/ **9** Refused [SMOK]

SMKP: **If No, what is your past smoking history?** **1** Former smoker/ **77** Never smoker/ **9** Refused or unsure [SMKP]

SMKS: **If you smoke, have you been advised by a health practitioner to stop smoking?**
1 [Does not smoke]/ **2** Yes (Advised to quit]/ **3** No/ **9** Refused or unsure [SMKS]

MEDICATIONS

ASPN: **Are you taking aspirin on daily basis?** **1** Yes/ **2** No/ **9** Refused [ASPN]

MED: **Are you currently taking any other pills or injections**? **1** Yes/ **2** No/ **9** Refused [MED]

IF YES, may I see the pills and injections you use and ask a few questions about them?
Note: Have the respondent show you all the pills, insulin, and other medicines that he or she is currently taking. Fill in the chart for each of these.

**COMPOUND (MEDICINE), OR TYPE OF INSULIN, OR TYPE OF INSULIN SYRINGE OR PEN (IF NOT PRE-FILLED WITH INSULIN)**	**MG PER PILL OR INSULIN UNITS TAKEN PER DAY**	**# PILLS OR SHOTS PRE-SCRIBED PER DAY**	**# PILLS/ SHOTS TAKEN PER DAY, (WHEN TAKEN)**	**# DAYS/WEEK PILLS/ SHOTS TAKEN**<br>1. EVERY DAY<br>2. 4-6 DAYS<br>3. 1-3 DAYS<br>4. < 1/WEEK	**IF NOT TAKEN AS PRESCRIBED WHY NOT (PRIMARY REASON)**<br>0. AS PRESCRIBED<br>1. SIDE EFFECTS<br>2. CANNOT AFFORD<br>3. FORGET<br>4. HARD TO GET<br>5. HELP NOT AVAILABLE<br>6. SYRINGE EQUIPMENT PROBLEMS<br>7. DON'T BELIEVE IT WORKS<br>88. DON'T NEED IT<br>8. DON'T KNOW<br>9. REFUSED	**WHERE WAS THE MEDICATION OBTAINED?**<br>1. PRIVATE/PHARMACY<br>2. HOSPITAL PHARMACY<br>3. CLINIC<br>4. PUBLIC OR STREET MARKET<br>5. RELATIVE OR FRIEND<br>66. OTHER<br>8.DON'T KNOW<br>9. REFUSED	**HOW MUCH DID YOU PAY THE LAST TIME YOU BOUGHT THIS MEDICINE?**<br>**[IF COST OF MEDICINE WAS PART OF DOCTOR OR HOSPITAL FEE, ENTER 999]**	**HOW MANY PILLS OR UNITS OF INSULIN DID YOU GET?**
M1A	M1B	M1C	M1D	M1E	M1F	M1G	M1H	M1I
M2A	M2B	M2C	M2D	M2E	M2F	M2G	M2H	M2I

M3A	M3B	M3C	M3D	M3E	M3F	M3G	M3H	M3I
M4A	M4B	M4C	M4D	M4E	M4F	M4G	M4H	M4I
M5A	M5B	M5C	M5D	M5E	M5F	M5G	M5H	M5I
M6A	M6B	M6C	M6D	M6E	M6F	M6G	M6H	M6I
M7A	M7B	M7C	M7D	M7E	M7F	M7G	M7H	M7I
M8A	M8B	M8C	M8D	M8E	M8F	M8G	M8H	M8I
M9A	M9B	M9C	M9D	M9E	M9F	M9G	M9H	M9I

QUESTIONS FOR PERSONS WITH DIABETES

(If respondent does not have diabetes, skip to ETIM and conclude the interview.)

|___|___| Yrs |___|___| Months DM2

DM2: **For how many years have you had diabetes?** 99 if refuses or cannot make any estimate

DM3: **During the LAST 90 DAYS, did you test your own blood sugar?**
1 Yes (**CONTINUE TO DM3AA**)/ **2** No (**GO TO DM4**)/ **9** Refused — DM3

DM3AA: **During the LAST 90 DAYS, how often did you test your own blood sugar?**
1 More than twice a day/ **2** Twice a day**/ 3** Once a day/ **4** Once or twice a week/ **5** Less than once a week/ **6** Once a month/ **7** Once in 3 months — DM3AA

DM3A: **How many blood sugar testing strips did you receive, the last time you got some?**
Use leading zeros. /**666 do not do home testing (GO TO DM4)** /**8 D**on't know/ **9** refused — DM3A

DM3B: **How much did you pay for these blood sugar testing strips?**
Use leading zeros /**666 do not do home testing** (**GO TO DM4)** /**8** don't know**/ 9** Refused — DM3B

DM3C: Where did you get these testing strips?
1 Private doctor or clinic / **2** Public doctor or clinic **/ 3** Private pharmacy or dispensary / **4** Other / **8** don't know**/ 9** Refused

DM3C

DM3D: Over the last year, have you been able to get all the testing strips you needed, when you needed them?
1 Yes, always/ **2** Most of the time**/ 3** Usually not/ **55** Never

DM3D

DM4: During the LAST 90 DAYS, how often has someone else measured your blood sugar, not counting tests your family did for you?
1 More than twice a day/ **2** Twice a day**/ 3** Once a day/ **4** Once or twice a week/ **5** Less than once a week/**6** Once a month/ **7** Once in 3 months/ **55** Never

DM4

DM5: When the cost of blood sugar test is separate from hospitals, clinics or doctors visits cost, how much do you usually pay to have someone perform one blood sugar test?
Use leading zeros / **666** no test performed /**8** don't know**/ 9** refused

Payment per test:
|__|__|__|__|__|__| DM5

DM6: Have you injected insulin at home or in a nearby clinic during the last 90 days?
1 Yes, I inject myself at home **(go to DM6H)**/ **2** Yes, I get shots at a clinic **(go to ETIM)**/ **3** No **(go to DM6A)**/ **8** Don't know/ **9** Refused.

DM6

DM6A: What are your main reasons for not using insulin?
1 Yes / **2** No / **8** Don't know/ **9** Refused

Reasons for not using insulin (Then go to ETIM)	Response
Insulin not prescribed by my doctor	INS1
Insulin too expensive—I cannot afford to buy it	INS2
Insulin not available or in stock in pharmacy or clinic	INS3
Insulin too weak, too strong, or adulterated (poor quality)	INS4
Syringes not in stock in pharmacy or clinic near my home	INS5
Syringes too expensive—I cannot afford to buy them	INS6
Afraid of or dislike needles	INS7
Don't how to inject insulin properly	INS8
Don't know when to inject insulin	INS9
Doctor prescribed it but I don't think I need it	INS10
Cannot find or afford blood sugar testing supplies	INS11
Doctor or nurse not available in the clinic	INS12
Other reason	INS13
Don't know or refused	INS14

DM6H: **During the last 90 days, on how many days did you fail to inject your insulin because you did not have usable syringes or needles?** **1** never/ **2** 5 days or fewer/ **3** 6-10 days/ **4** 11-20 days/ **5** 21-45 days/ **6** more than half the time/ **7** always (did not inject insulin last 90 days because did not have syringes)/ **8** don't know/ **9** refused.

DM6H

DM6I: **During the last 90 days, about how many times did you re-use each needle, to give yourself insulin?** **1** never (I always used a new needle for each injection)/ **2** Twice/ **3** three times/ **4** four times/ **5** five times or more/ **8** don't know/ **9** refused

DM6I

ETIM: **Ending time:**
24 hour clock

|__|__| Hour |__|__| Minutes ETIM
